# Comparative analysis of the Rotarix™ vaccine strain and G1P[8] rotaviruses detected before and after vaccine introduction in Belgium

**DOI:** 10.7717/peerj.2733

**Published:** 2017-01-03

**Authors:** Mark Zeller, Elisabeth Heylen, Sana Tamim, John K. McAllen, Ewen F. Kirkness, Asmik Akopov, Sarah De Coster, Marc Van Ranst, Jelle Matthijnssens

**Affiliations:** 1 Department of Microbiology and Immunology, Katholieke Universiteit Leuven, Leuven, Belgium; 2 Department of Microbiology, Quaid-i-Azam University, Islamabad, Pakistan; 3 The J. Craig Venter Institute, Rockville, MD, USA; 4 Human Longevity, Inc., San Diego, CA, USA

**Keywords:** Rotaviruses, Wa-like, G1P[8], Genetic diversity, Vaccine introduction

## Abstract

G1P[8] rotaviruses are responsible for the majority of human rotavirus infections worldwide. The effect of universal mass vaccination with rotavirus vaccines on circulating G1P[8] rotaviruses is still poorly understood. Therefore we analyzed the complete genomes of the Rotarix™ vaccine strain, and 70 G1P[8] rotaviruses, detected between 1999 and 2010 in Belgium (36 before and 34 after vaccine introduction) to investigate the impact of rotavirus vaccine introduction on circulating G1P[8] strains. All rotaviruses possessed a complete Wa-like genotype constellation, but frequent intra-genogroup reassortments were observed as well as multiple different cluster constellations circulating in a single season. In addition, identical cluster constellations were found to circulate persistently over multiple seasons. The Rotarix™ vaccine strain possessed a unique cluster constellation that was not present in currently circulating G1P[8] strains. At the nucleotide level, the VP6, VP2 and NSP2 gene segments of Rotarix™ were relatively distantly related to any Belgian G1P[8] strain, but other gene segments of Rotarix™ were found in clusters also containing circulating Belgian strains. At the amino acid level, the genetic distance between Rotarix™ and circulating Belgian strains was considerably lower, except for NSP1. When we compared the Belgian G1P[8] strains collected before and after vaccine introduction a reduction in the proportion of strains that were found in the same cluster as the Rotarix™ vaccine strain was observed for most gene segments. The reduction in the proportion of strains belonging to the same cluster may be the result of the vaccine introduction, although natural fluctuations cannot be ruled out.

## Introduction

Rotavirus A (RVA) is the most important etiological agent for diarrhea in children under 5 years of age worldwide (Tate et al., 2012). The eleven-segmented double stranded RNA genome allows rotaviruses to reassort frequently and aides to establish new variants of genes in the human RVA population. Rotaviruses are classified according to their outer capsid proteins VP7 and VP4, which determine the G- and P-genotype, respectively. In humans, the most common genotypes are G1P[8], G2P[4], G3P[8], G4P[8], G9P[8] and G12P[8] (Bányai et al., 2012). An extension of this dual classification system comprises all eleven segments and revealed the existence of two major genotype constellations in humans, often referred as Wa-like and DS-1-like (Matthijnssens et al., 2008). P[8] genotypes are commonly associated with a Wa-like genotype constellation (e.g., G1-P[8]-I1-R1-C1-M1-A1-N1-T1-E1-H1) and P[4] genotypes are commonly associated with a DS-1-like genotype constellation (e.g., G2-P[4]-I2-R2-C2-M2-A2-N2-T2-E2-H2). In humans, the Wa-like genotype constellation is the most important and over 90% of all infections are caused by rotaviruses belonging to this genotype constellation (Bányai et al., 2012; Matthijnssens & Van Ranst, 2012).

Within the Wa-like genogroup, G1P[8] genotypes are the most prevalent worldwide, although regional and temporal variations are common (Bányai et al., 2012). Therefore, one of the currently available rotavirus vaccines, Rotarix™, contains a live attenuated G1P[8] rotavirus. Rotarix™ has been available for use in Belgium since June 2006 and a very high coverage of approximately 90% was reached within months after vaccine introduction (Zeller et al., 2010). RotaTeq™ has been available in Belgium since June 2007 and is used less frequently (approximately 15% of all administered rotavirus vaccines is RotaTeq™) (Zeller et al., 2010; Matthijnssens et al., 2014). The genotype distribution of rotaviruses detected at the Gasthuisberg university hospital in Leuven has been studied since the 1999–2000 rotavirus season. During this period G1P[8] strains were detected in every season, although the prevalence of G1P[8] strains varied widely (Zeller et al., 2010). The genetic variability within the human G1 and P[8] genotypes is relatively large, especially when compared to other human genotypes and multiple G1 and P[8] lineages have been identified, although the identification of sub-genotypic lineages often occurs on an ad hoc basis (Parra et al., 2005; Matthijnssens et al., 2010; Zeller et al., 2012).

It has previously been shown that routine vaccination with Rotarix™ is associated with an increased proportion of fully heterotypical G2P[4] rotaviruses (Gurgel et al., 2007; Zeller et al., 2010; Matthijnssens et al., 2014). Less is known on whether the introduction of Rotarix™ also selects for certain lineages within the G1P[8] genotype and how this effects the overall genetic diversity of G1P[8] strains. In general, rotavirus evolution is poorly understood and is determined by the accumulation of point mutations over time and by reassortment events. The accumulation of point mutations results in genetic and antigenic drift, whereas reassortment of gene segments allows for sudden adaptations to altering selection pressures, probably playing an important role in viral evolution and the generation of genetic diversity. Gene reassortment is not a random process and is restricted by structural and functional viral protein-protein interactions and host specificity. In humans, this is reflected in the existence of only two major genotype constellations (Matthijnssens & Van Ranst, 2012). Therefore, viral fitness is most often maintained or increased when a gene reassortment occurs among gene segments belonging to the same (lineages of a) genotype. Precisely how often reassortment occurs in human rotaviruses, and what factors are involved, is poorly understood as many surveillance efforts tend to primarily focus on the VP7 and VP4 gene segments (Iturriza-Gómara et al., 2011; Mwenda et al., 2014).

Large scale comparative studies of Wa-like rotaviruses in the United States revealed frequent intra-genogroup reassortment, but no inter-genogroup reassortment was observed (McDonald et al., 2009; McDonald et al., 2011; McDonald et al., 2012). In contrast, persistent genotype constellations were also observed to circulate in multiple seasons, suggesting that even within a single genotype constellation preferred constellations may be present. However, none of these studies specifically investigated the effect of vaccine introduction on the intra-genotype dynamics of commonly circulating Wa-like strains. Previously, we have reported the G1P[8] strain diversity in Belgium and Australia, and showed the existence of unique subclusters that were present only after vaccine introduction (Zeller et al., 2015). In this study we investigate 70 Belgian G1P[8] strains in relationship with the Rotarix™ vaccine. This allowed us to analyze how circulating G1P[8] strains collected before and after vaccination relate to the Rotarix™ vaccine strain, and will contribute to our understanding of the impact of vaccination on the genetic diversity of rotaviruses, particularly G1P[8] strains.

## Methods

### Sample collection and sequencing of wild-type G1P[8] rotaviruses

In total 70 G1P[8] RVA strains were selected based on their phylogenetic clustering of VP7 in such a way that the selection reflected the genetic diversity of G1P[8] strains in Belgium. For each sample approximately 50 mg of collected feces was resuspended in 500 µl PBS and viral RNA was subsequently extracted with the Qiagen Viral RNA minikit (Qiagen) according to the manufacturer’s instructions. In total 100 µl of viral RNA was sent to the J. Craig Venter Institute for RT-PCR and sequencing in a high-throughput fashion as described previously (McDonald et al., 2009; Zeller et al., 2015). A Rotarix™ G1P[8] vaccine strain was commercially obtained in Belgium to compare the vaccine strain with circulating RVAs in Belgium, and reconstituted according to manufacturer’s recommendations (lot number: A41CB052A). Viral RNA was extracted with the Qiagen Viral RNA minikit (Qiagen) according to manufacturer’s instructions and subsequently the VP1-VP3, VP6 and NSP1-NSP5 gene segments were amplified using the primers listed in Table S1 with a one-step RT-PCR kit (Qiagen). The nucleotide sequences of Rotarix™ VP7 and VP4 gene segments have been determined previously (Zeller et al., 2012). The obtained amplicons were subsequently pooled in a ratio dependent on the length of each gene segment and sequenced using 454™ GS-FLX pyrosequencing as previously described (Heylen et al., 2015). Obtained reads were mapped against G1P[8] reference strain Wa using MIRA 3.4 (Chevreux et al., 2004) and assemblies were visually inspected in Tablet (Milne et al., 2013). Subsequently the consensus sequences for 9 gene segments of Rotarix™ were determined, used for further analysis and deposited in GenBank under accession numbers: KX954616– KX954624.

### Nucleotide sequence analysis

The genotypes for each of the eleven gene segments were determined using the online rotavirus genotyping tool, RotaC (http://rotac.regatools.be) (Maes et al., 2009). Sequences were aligned and the most optimal nucleotide substitution model was determined in Mega 5 (Tamura et al., 2011). Maximum likelihood phylogenetic tree construction was performed using the GTR substitution model allowing for a gamma-distributed rate variation among sites with 500 bootstrap replicates. These maximum likelihood trees were subsequently used to automatically detect clusters with the CTree heuristic cluster finding algorithm using the most sensitive parameters (Archer & Robertson, 2007). This method takes into account the overall genetic diversity of a gene segment when partitioning the sequence data into clusters. Matlab was used to calculate and plot genetic differences between Rotarix™ and circulating G1P[8] strains. Statistical differences in prevalence of lineages circulating before and after vaccine introduction were determined by Fisher’s exact test.

## Results

### Viral dataset

Belgian G1P[8] stool samples were collected between 1999 and 2010 from hospitalized children with acute gastroenteritis. Until the 2006–2007 season, when no vaccines were available in Belgium, G1P[8] was the most prevalent genotype. After the 2006–2007 season, when the Rotarix™ vaccine was introduced, G2P[4] was the most prevalent genotype and G1P[8] the second most prevalent (Zeller et al., 2010). In total 36 G1P[8] strains were collected before vaccine introduction and 34 were collected after vaccine introduction according to the selection criteria described above. For every gene segment of all 70 Belgian G1P[8] strains, the genotype was determined using the RotaC genotyping tool and a complete Wa-like genotype constellation for all 70 Belgian G1P[8] strains was observed.

### Belgian G1P[8] rotaviruses display frequent intra-genotypic reassortment and persistent cluster constellations

**Figure 1:**
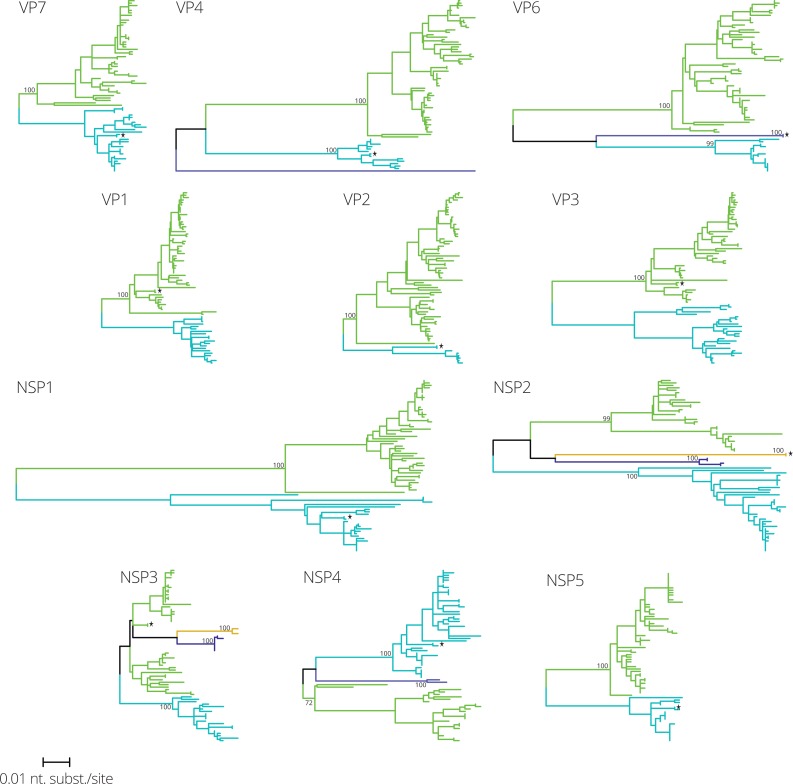
Maximum likelihood phylogenetic trees for eleven gene segments of 70 Belgian G1P[8] rotaviruses and the Rotarix™ vaccine strain, which is indicated by a black star. All trees are drawn to the same scale and phylogenetic trees were divided in one to four clusters colored in green, blue, purple and orange, respectively.

To determine intra-genotypic variation and reassortment patterns the 70 G1P[8] strains together with the Rotarix™ vaccine strain were used for maximum likelihood phylogenetic tree reconstruction. For each gene segment phylogenetic clusters were assigned using the automated cluster finding algorithm implemented in Ctree (Archer & Robertson, 2007) (Fig. 1 and Fig. S1). Most gene segments were divided into two clusters (VP7, VP1, VP2, VP3, NSP1 and NSP5), whereas other gene segments were partitioned in three (VP4, VP6 and NSP4) or four clusters (NSP2 and NSP3). The genetic distance between clusters was largest for NSP1, NSP2, VP4 and VP6. One Belgian G1P[8] strain, RVA/Human-wt/BEL/BE00048/2009/G1P[8], was almost identical to Rotarix™ for every gene segment. Together with strain BE00048, the Rotarix™ vaccine strain was found in similar clusters as Belgian G1P[8] strains, except for VP6 and NSP2, for which Rotarix™ and BE00048 constituted separate clusters.

**Figure 2:**
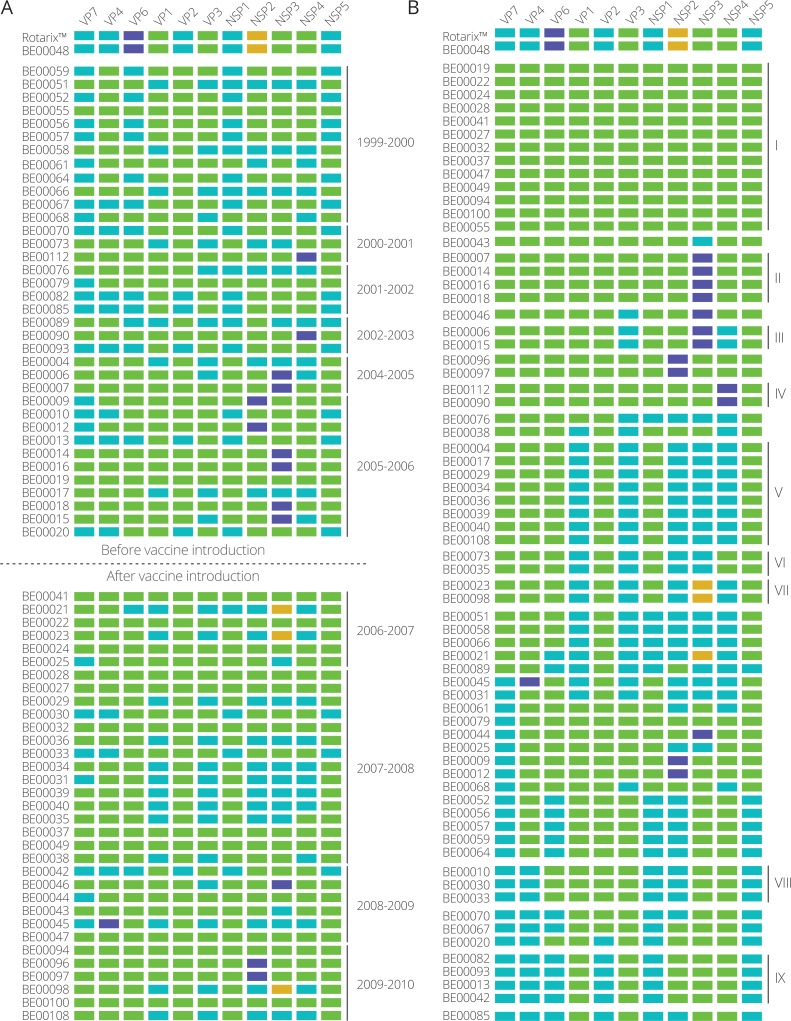
Cluster constellations of 70 Belgian G1P[8] rotaviruses and the Rotarix™ vaccine strain. For every strain, each gene segment is assigned in a cluster indicated by green, blue, purple or orange. (A) Cluster constellations chronologically ordered. The vaccine introduction is indicated by a dashed line. (B) Cluster constellations ordered by similarity. Persistent cluster constellations over multiple seasons are indicated by roman numerals.

The identification of clusters within each gene segment allowed us to determine the cluster constellation for each of the Belgian G1P[8] rotaviruses and the Rotarix™ vaccine strain (Fig. 2A). In every rotavirus season G1P[8] rotaviruses with different cluster constellations were co-circulating. This was the case for rotavirus seasons before vaccine introduction as well as after vaccine introduction. During the study period also the introduction of novel G1P[8] clusters was observed such as the purple clusters in NSP2, NSP3 and NSP4 (Fig. 2A). Most of them were only minor variants and tended to circulate for only a limited number of seasons before disappearing and re-emerging in later rotavirus seasons. For all gene segments there were only two major clusters (indicated in green and blue) that were circulating throughout the whole study period.

When the 70 G1P[8] strains were ordered according to their cluster constellation similarity, 30 different cluster constellations were identified (Fig. 2B). However, only nine cluster constellations (I–IX) were found to persistently circulate in multiple rotavirus seasons and these ranged in size from two strains (cluster constellation III, VI and VII) to up to twelve strains (cluster constellation I). The longest circulating clusters, cluster I and V, were circulating in seven and four rotavirus seasons, respectively. Cluster I was first observed in 1999–2000, reappeared in 2005–2006 and was found in all subsequent rotaviruses seasons, whereas cluster V was detected in 2004–2005, 2005–2006, 2007–2008 and 2009–2010. Other persistent clusters consisted of fewer strains and were either found in successive rotavirus seasons (cluster II and III) or were found multiple rotavirus seasons apart (cluster IV, VI, VII, VIII and IX).

The Rotarix™ vaccine strain possessed a cluster constellation that was not found in other circulating Belgian G1P[8] strains, except for BE00048, which possessed an identical cluster constellation to that of Rotarix™ and was obtained from a child that was vaccinated approximately 10 weeks before the sample was collected and was almost certainly vaccine-derived (Zeller et al., 2012). The Rotarix™ and BE00048 cluster constellations were found to possess unique clusters for the VP6 and NSP2 gene segment (Fig. 2; purple and orange clusters for VP6 and NSP2, respectively). Although BE00048 and Rotarix™ contained similar cluster constellations, 18 nucleotide differences were observed (Table 1). These differences were found in the VP7, VP4, VP6, VP1, VP3, NSP1, NSP4 and NSP5 gene segments and except for two nucleotide changes in VP4 and NSP1, they all resulted in amino acid changes. However, base calling was ambiguous at many of these nucleotide positions, indicating that both the original nucleotide of the vaccine strain as well as the novel variant were present at a particular position.

**Table 1:** Nucleotide and amino acid differences between the Rotarix™ vaccine strain and RVA/Human-wt/BE00048/2009/G1P[8].

Gene segment	Nucleotide change	Amino acid change
VP7	T605C	M202T
VP4	T501W	F167?
	C1175M	A392?
	C1515T	–
VP6	T632Y	I211?
	G634R	V212?
VP1	T90Y	S30?
	G1426A	V476I
VP3	C319T	H107Y
	T937C	S313P
	C1838T	A613V
NSP1	A15G	–
NSP4	G109A	A37T
	T125K	V42?
	T128Y	L43?
	T137Y	L46?
NSP5	G273R	M91?
	G274T	D92Y

**Figure 3:**
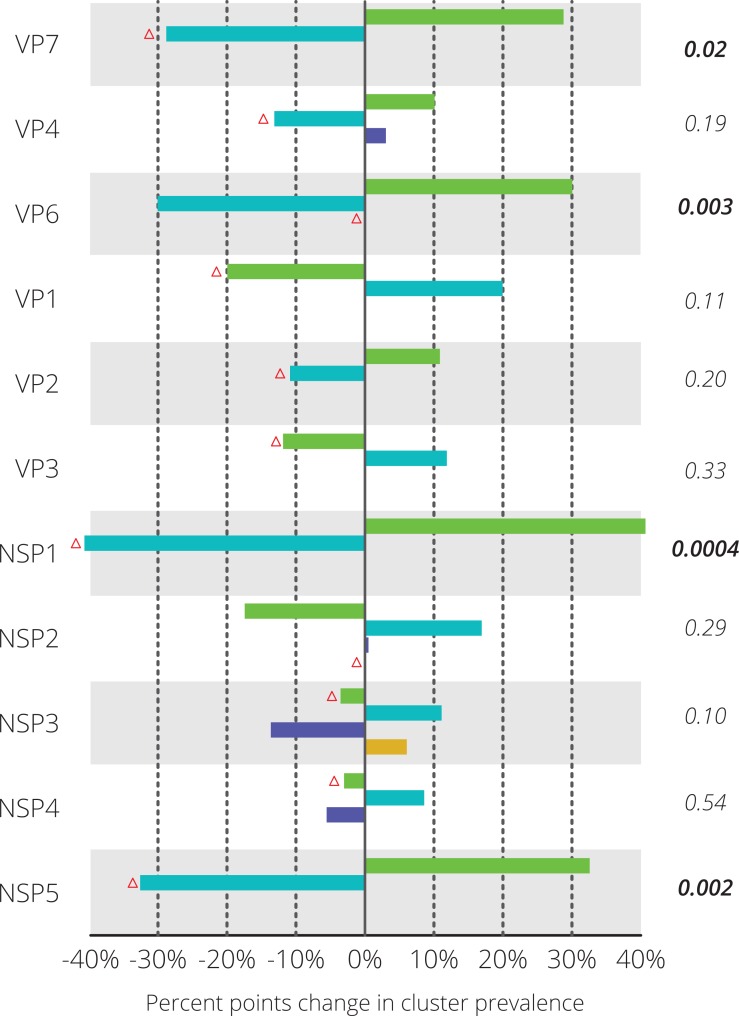
Differences in relative prevalence of clusters before and after vaccine introduction. For every gene segment the cluster containing Rotarix™ is indicated with a red triangle. Statistical differences were tested using Fisher’s exact test and the resulting *p*-value is shown on the right-hand side. Significant *p*-values are indicated in bold face.

**Figure 4:**
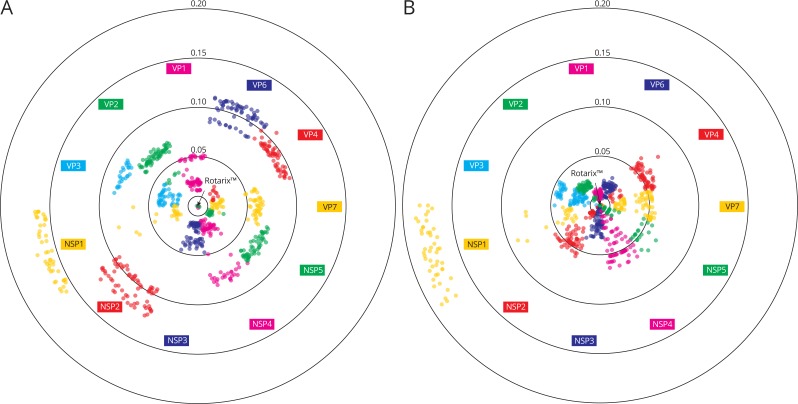
Genetic distances between 70 Belgian G1P[8] rotavirus strains and the Rotarix™ vaccine strain on the nucleotide level (A) and on the amino acid level (B). Rotarix™ is positioned in the center and each wild-type strain is represented with a filled circle. A higher genetic distance to Rotarix™ is indicated by a more outward position.

### G1P[8] clusters containing the vaccine strain were less prevalent after vaccine introduction

For each gene segment the prevalence of different clusters before and after vaccine introduction was determined (Fig. 3). Statistically significant differences in prevalence before and after vaccine introduction were observed in the NSP1 (40.6%), NSP5 (32.6%), VP7 (28.8%) and VP6 (30.1%) gene segments. For these four gene segments, blue colored clusters became less prevalent after vaccine introduction when compared to seasons before vaccine introduction. For nine out of eleven gene segments, the Rotarix™ vaccine strain belonged to the cluster that was relatively less prevalent after vaccine introduction. For VP6 and NSP2 the Rotarix™ vaccine strain belonged to a unique cluster and no changes in relative prevalence could be determined.

To gain a more detailed insight in how closely Belgian G1P[8] rotaviruses were related to the Rotarix™ vaccine strain the genetic distance of 70 Belgian G1P[8] strains to Rotarix™ was determined for every gene segment (Fig. 4A). At the nucleotide level, large differences were observed between the different gene segments. The maximum genetic distance to Rotarix™ was largest for NSP1 (17.0%), followed by NSP2 (12.1%) and VP6 (11.3%). NSP3, VP1 and VP7 were most closely related to Rotarix™ with a maximum distance of 4.7%, 5.3% and 6.5%, respectively. For most gene segments the genetic distance to Rotarix™ was characterized by groups of strains with a relatively low genetic distance (generally less than 5 percent) and groups of strains with a higher genetic distance to Rotarix™, reflecting the different clusters as defined previously. For the VP6 and NSP2 gene segments, all circulating G1P[8] strains were relatively distantly related to the vaccine strain, the most closely related strain to Rotarix™ were 8.6% and 9.4% different, respectively.

In general, the genetic distances to the Rotarix™ vaccine strain were lower at the amino acid level than at the nucleotide level with exception of NSP1, for which the maximum genetic diversity increased from 17.0% at the nucleotide level to 18.5% at the amino acid level (Fig. 4B). The VP1 gene segment showed the highest similarity between Rotarix™ and circulating Belgian G1P[8] strains at the amino acid level (1.7%). The VP6 and NSP2 gene segment, which were relatively distantly related to Rotarix™ at the nucleotide level, were relatively closely related to the vaccine strain at the amino acid level (3.0% and 5.4%, respectively) and 91.4% and 85.9% of all nucleotide differences with the vaccine strain were synonymous, respectively.

## Discussion

Rotavirus vaccines have been introduced in many countries around the world (Patel et al., 2012), but rotaviruses still remain an important cause of morbidity and mortality. Despite this, our knowledge of their genetic diversity is still relatively limited compared to other viruses such as influenza A virus, and has considerably hindered our understanding of rotavirus genetic diversity and of how rotaviruses evolve between consecutive seasons. In particularly, it is unknown what the impact of vaccine introductions are on rotavirus evolution.

In the present study the effect of rotavirus vaccine introduction on circulating G1P[8] rotaviruses was investigated. Previously, we have shown in a large comparative study comprising Belgian and Australian RVA strains that gene segments probably have distinct evolutionary histories and that unique phylogenetic subclusters were present after vaccine introduction (Zeller et al., 2015). Here we show by using the same G1P[8] sample collection that depending on the gene segment, substantial differences exist in comparison with the Rotarix™ vaccine strain and that non-Rotarix™ clusters were more prevalent after vaccine introduction.

G1P[8] is worldwide the most prevalent genotype and also in Belgium G1P[8] is one of the few genotypes that was observed in all RVA seasons since rotavirus surveillance started in 1999 (Zeller et al., 2010; Bányai et al., 2012). Rotavirus vaccination in Belgium was introduced in 2006 and within two rotavirus seasons reached a high coverage of approximately 85% (Zeller et al., 2010; Braeckman et al., 2011). Uniquely, our dataset comprised rotavirus samples spanning six seasons before and four seasons after vaccine introduction. Although only Wa-like genotypes were found, a relative high genetic diversity was observed at the subgenotype level. This diversity was present in all eleven gene segments and was used to divide our dataset into clusters, revealing a high level of reassortment among G1P[8] strains in Belgium. Previously, this was also shown in an archival dataset containing 51 G3P[8] RVAs and in a contemporary dataset comprising 58 G1P[8], G3P[8] and G12P[8] strains (McDonald et al., 2009; McDonald et al., 2012). These samples were collected in the USA in a period without any use of rotavirus vaccines (McDonald et al., 2009) or in a transition period where the first season of sample collection was before vaccine introduction while during the subsequent three seasons the RotaTeq™ vaccine was widely used (McDonald et al., 2012). The findings of a high intra-genotype reassortment frequency in combination with a significant genetic diversity in this study, where a balanced set of G1P[8] samples before and after vaccine introduction was obtained, suggests that this is a typical and apparently widely occurring characteristic of rotavirus epidemiology over an extended period of time.

Until now it was unclear if universal mass vaccination with any of the two currently available rotavirus vaccines will affect this pattern. In our dataset, however, we did find the occurrence of intra-genotype reassortment both before and after vaccine introduction. In contrast, we also found that certain subtypes were less prevalent after vaccine introduction and thereby reducing the possibility of intra-genotype reassortment. Because Belgium is a relatively small country and in neighboring countries of Belgium rotavirus vaccines are not widely used, a part of the genetic diversity within G1P[8] strains could also be (re-)imported from neighboring countries. In our dataset, we found clear evidence of emerging and reemerging minor variants as new clusters in for example NSP2 and NSP3. Thus, continuing surveillance involving complete genome sequencing will be essential to further expand our insights into these mechanisms.

Sequencing of the Rotarix™ vaccine strain did not reveal any differences with the Rotarix™ vaccine sequences previously deposited in GenBank (accession numbers: JX943604 –JX943614) and showed that Rotarix™ possessed a unique cluster constellation which was not found in wild-type Belgian circulating G1P[8] strains. A potential cause of this discrepancy could be the result of a 20–30 years difference in detection date between the parent virus of the Rotarix™ vaccine and circulating Belgian G1P[8] rotaviruses. Previously, it was shown that this resulted in numerous amino acid changes in antigenic sites of VP7 and VP4 (Zeller et al., 2012). Here we show that also for other gene segments, especially VP6, VP2 and NSP2, a considerable genetic distance exists between Rotarix™ and circulating strains. As the precise mechanism of protection afforded by Rotarix™ is unknown, it is possible that further accumulation of point mutations could over time result in a reduced effectiveness of the vaccine. However, the unique cluster constellation also represents a significant benefit with regard to diagnostics and detection of vaccine strains circulating in the human population. For example, a 11.3–12.1% nucleotide difference was observed between the VP6 and NSP2 gene segments of circulating G1P[8] strains and their Rotarix™ counterparts (Fig. 4A), turning them into primary targets to discriminate between vaccine-derived and wild-type G1P[8] rotaviruses. In fact, such an assay based on NSP2 was recently developed (Gautam et al., 2014). Although these Rotarix™ clusters were specific to the Rotarix™ vaccine in Belgium, we cannot rule out that in different parts of the world the VP6 or NSP2 clusters present in the vaccine are still in circulation. Furthermore, there are some indications that the NSP2 gene segment might reassort relatively easily as inter-genogroup reassortment has been more often reported for NSP2 than for many other gene segments including VP6 (Matthijnssens & Van Ranst, 2012). Thus, even if a vaccine-derived NSP2 is detected, sequencing additional gene segments is needed to exclude the possibility of reassortment between vaccine and wild-type strains.

Our dataset comprised one strain (BE00048) that was most likely completely vaccine-derived. Also in other countries vaccine-derived strains have been detected and even horizontal transmission between siblings has been described (Rivera et al., 2011; Donato et al., 2012; Hemming & Vesikari, 2012). Besides complete vaccine-derived strains, various reports of reassortment between vaccine strains and circulating strains have also been made (Bucardo et al., 2012; Rose et al., 2013). It is unclear on what scale this reassortment occurs however, but no evidence for reassortment was found in our dataset despite the extensive use of rotavirus vaccines in Belgium. The 18 nucleotide differences observed between BE00048 and the Rotarix™ vaccine could be either the result of *de novo* mutations or positive selection of mutations already present in the vaccine. Unfortunately, we were not able to confirm minor variants present in the vaccine as the sequence depth was low.

The selection of G1P[8] rotaviruses was mainly based on the phylogenetic diversity of VP7. Although a lot of effort was made to reflect the existing genetic diversity we cannot rule out certain biases. For example, no G1P[8] strains were selected from the 2003–2004 season even though G1P[8] strains were circulating in that particular season (Zeller et al., 2010). Also, selecting a subset of samples implies that we may not have been able to capture the full diversity of relatively rare subtypes within G1P[8]. However, the dataset provides a good overview of the diversity and prevalence of the more common subtypes (generally cluster I and cluster II) within G1P[8] rotaviruses circulating in Belgium.

To gain more insight in the long-term effects of vaccine introduction continued surveillance is necessary, including complete genome sequencing of rotaviruses on a routine basis. Sequence independent amplification in combination with next-generation sequencing techniques seems a powerful, inexpensive and relatively effortless tool to accomplish this.

##  Supplemental Information

10.7717/peerj.2733/supp-1 Table S1 Primers used to amplify all eleven gene segments of the Rotarix™ vaccine strain

10.7717/peerj.2733/supp-2 Figure S1 Enlargements of the maximum likelihood trees for all gene segments as used for Fig. 1. The color-codings of the tree branches indicate the different clusters. Bootstrap values are based on 500 replicates and only values above 70 are shown

10.7717/peerj.2733/supp-3 Supplemental Information 1 Sequences submitted to GenBank

## Additional Information and Declarations

### DNA Deposition

The following information was supplied regarding the deposition of DNA sequences: Sequences were deposited in GenBank under accession numbers KX954616–KX954624.

### Data Availability

The following information was supplied regarding data availability: The raw data or code is included in the manuscript.
