# Supplementary material for: Comparative analysis of the Rotarix™ vaccine strain and G1P[8] rotaviruses detected before and after vaccine introduction in Belgium
Source: PeerJ. 2017 Jan 3;5:e2733. doi: 10.7717/peerj.2733 (PMC5214804; doi:10.7717/peerj.2733)
Supplement: Table S1 [file peerj-05-2733-s001.pdf]

Gene segment	Primer name	Primer sequences
VP1	GEN_VP1Fb	5′-GGCTATTAAAGCTRTACAATGGGGAAG-3'
	GEN_VP1Rb	5′-GGTCACATCTAAGCGYTCTAATCTTG-3'
VP2	GEN_VP2Fc	5′-GGCTATTAAAGGYTCAATGGCGTACAG-3'
	GEN_VP2_Rbc	5′-GTCATATCTCCACARTGGGGTTGG-3'
VP3	GEN_VP3Fe	5′-GGCTWTTAAAGCARTATTAGTAGTG-3′
	GEN_VP3_2584R	5'-TGACYAGTGTGTTAAGTTTYTAGC-3'
VP4	VP4-1-17F	5′-GGCTATAAAATGGCTTCGC-3′
	GEN_VP4_P8_2328R	5'-CATTGTAGAATTARYTGTTCAATTCTATTCC-3'
VP6	GEN_VP6F	5′-GGCTTTWAAACGAAGTCTTC-3
	GEN_VP6R	5′-GGTCACATCCTCTCACT-3′
VP7	BEG9	5′-GGCTTTAAAAGAGAGAATTTCCGTCTGG-3′
	END9	5′-GGTCACATCATACAATTCTAATCTAAG-3′
NSP1	GEN_NSP1F	5′-GGCTTTTTTTTATGAAAAGTCTTG-3′
	GEN_NSP1R	5′-GGTCACATTTTATGCTGCC-3′
NSP2	GEN_NSP2F	5′-GGCTTTTAAAGCGTCTCAG-3′
	GEN_NSP2R	5′-GGTCACATAAGCGCTTTC-3′
NSP3	GEN_NSP3F	5′-GGCTTTTAATGCTTTTCAGTG-3′
	GEN_NSP3R	5′-ACATAACGCCCCTATAGC-3′
NSP4	GEN_NSP4F	5′-GGCTTTTAAAAGTTCTGTTCC-3′
	GEN_NSP4R	5′-GGWYACRYTAAGACCRTTCC-3′
NSP5	GEN_NSP5F	5′-GGCTTTTAAAGCGCTACAG-3′
	GEN_NSP5R	5′-GGTCACAAAACGGGAGT-3′
