# Supplementary figures and images for: Comparative analysis of the Rotarix™ vaccine strain and G1P[8] rotaviruses detected before and after vaccine introduction in Belgium
Source: PeerJ. 2017 Jan 3;5:e2733. doi: 10.7717/peerj.2733 (PMC5214804; doi:10.7717/peerj.2733)

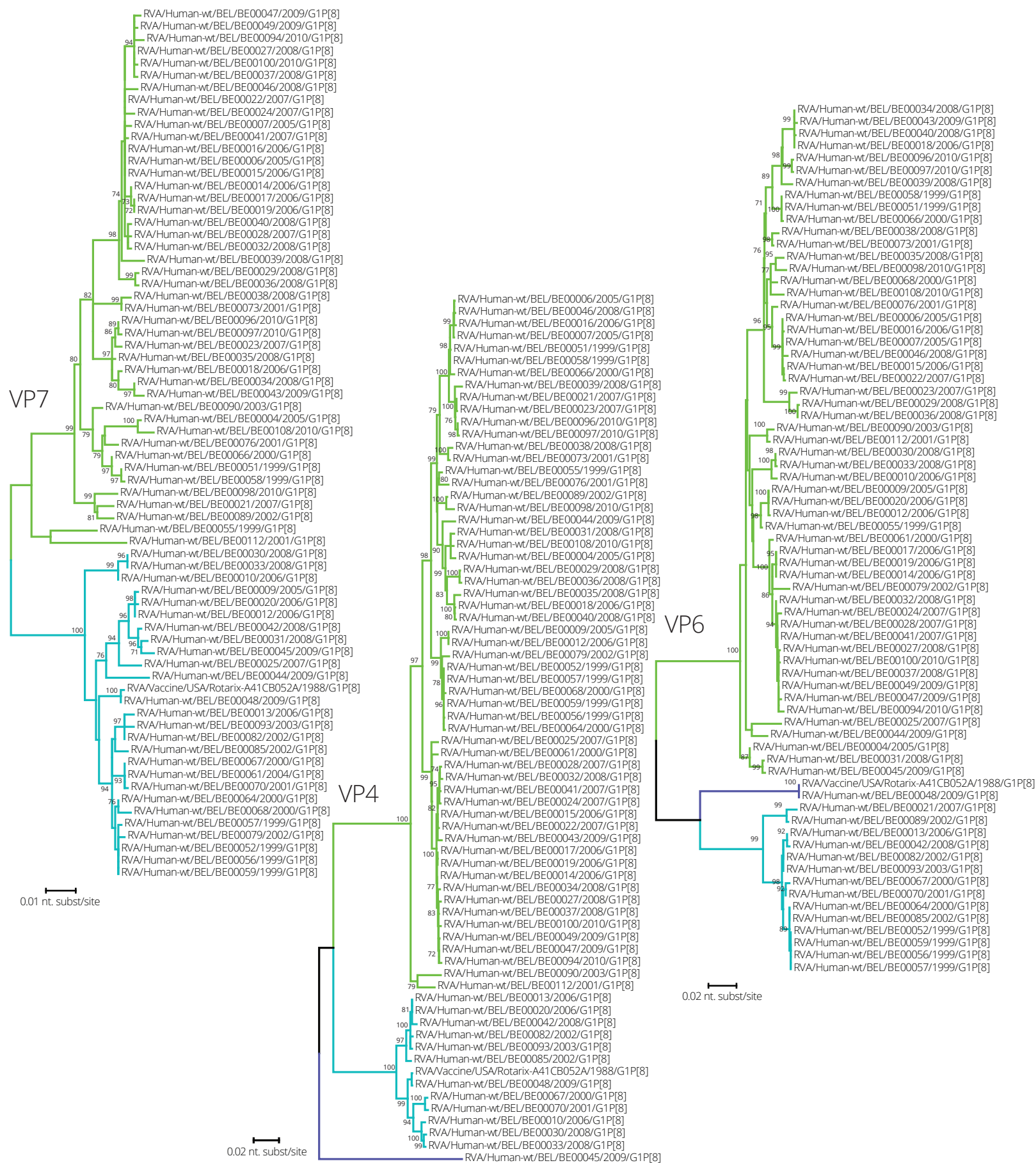

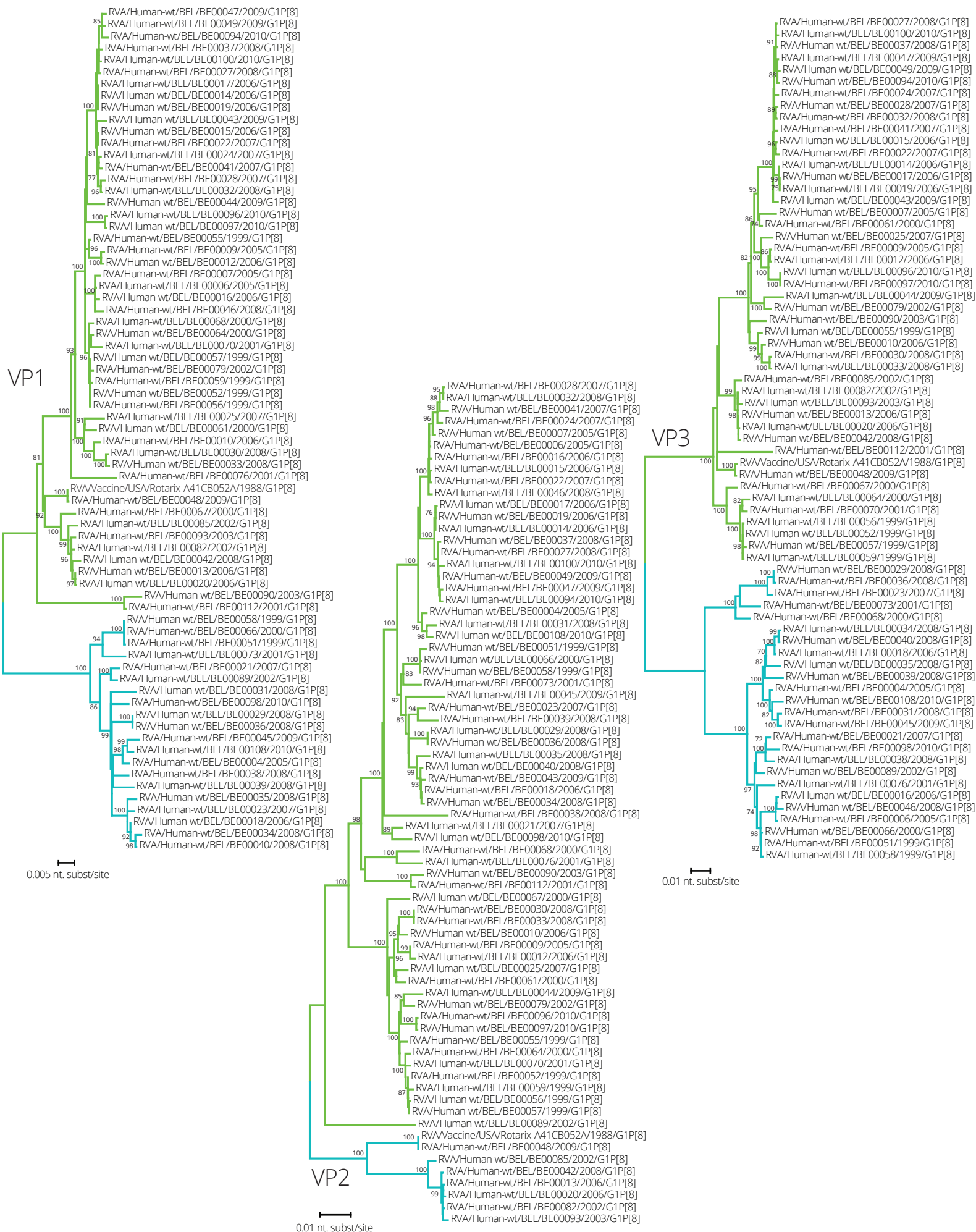

NSP1

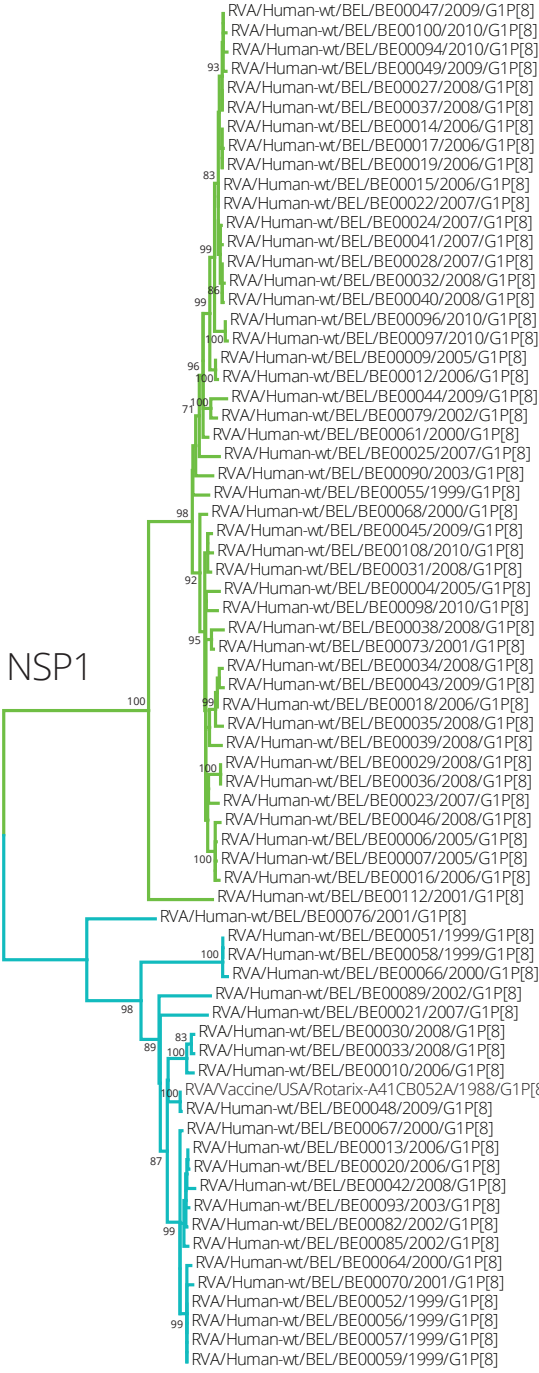

NSP2

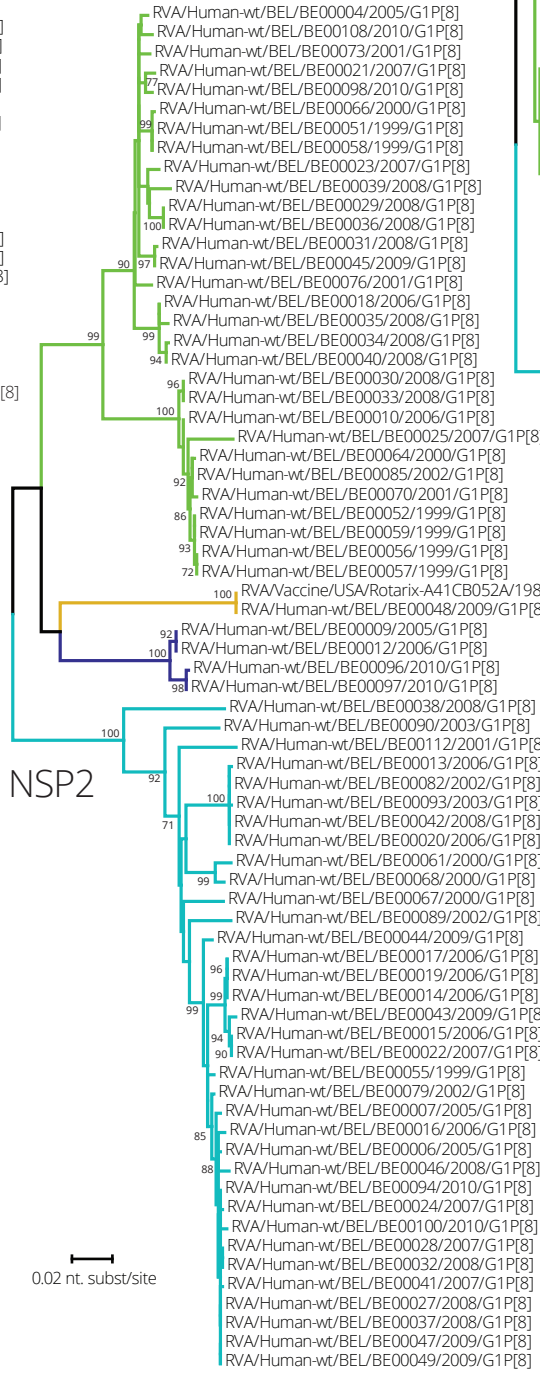

NSP3

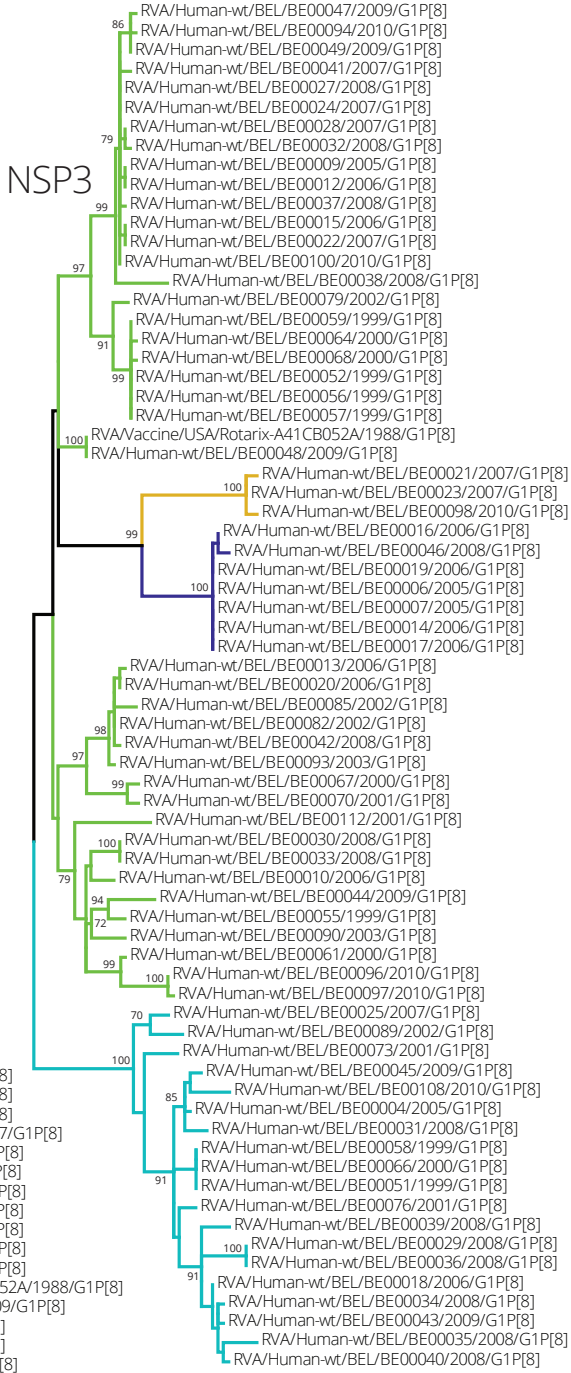

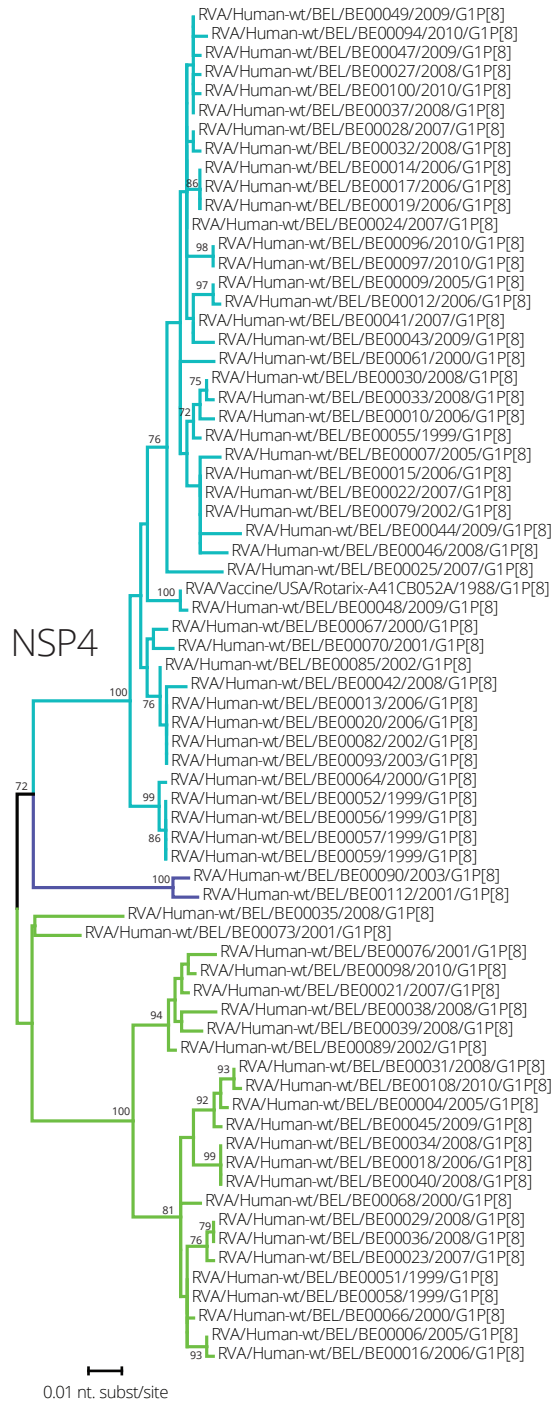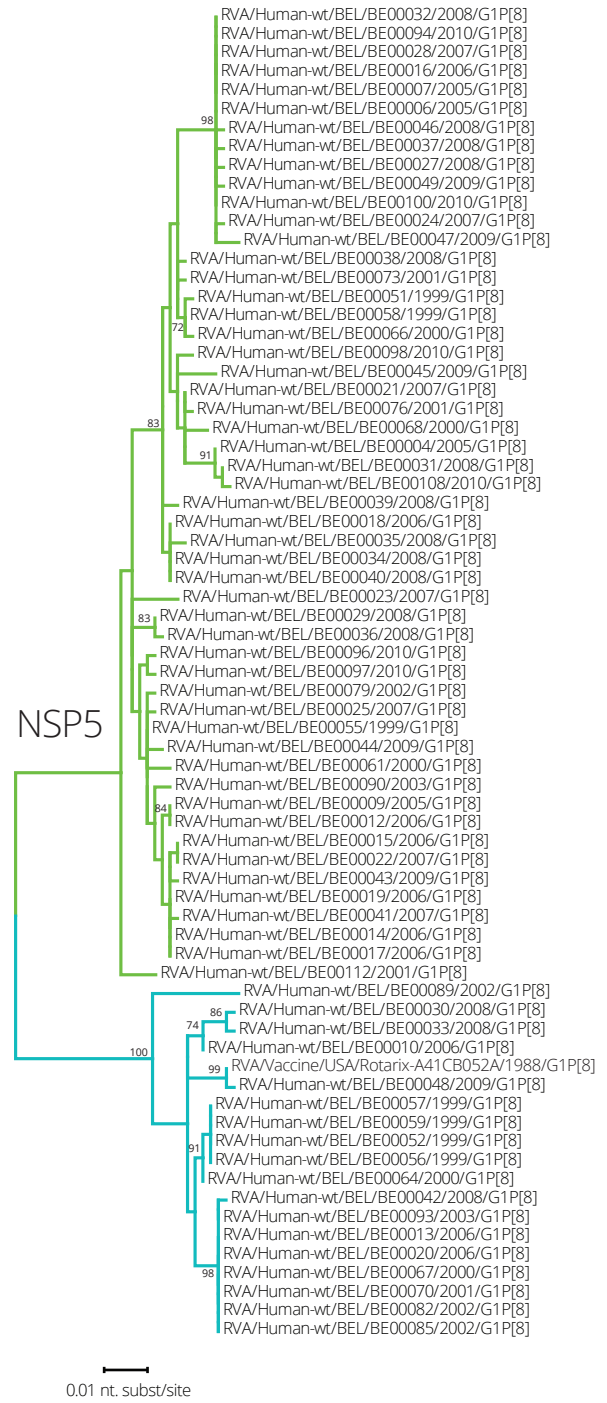

Supplement: Figure S1 [file peerj-05-2733-s002.pdf]
